# Heart failure in 2015: let’s get organised!

**DOI:** 10.1007/s12471-015-0722-5

**Published:** 2015-06-04

**Authors:** C.M.H.B. Lucas, P.E.J. van Pol, J.B.E. Eysink Smeets, M. Niesing, H.F. Verwey, S.L.M.A. Beeres

**Affiliations:** 1 Department of Cardiology, Alrijne Hospital, Leiderdorp, The Netherlands; 2 Huisartspraktijk Prelude, Alphen aan den Rijn, The Netherlands; 3 Department of Cardiology, Leiden University Medical Center, Albinusdreef 2, 2333 ZA Leiden, The Netherlands

Heart failure represents a serious challenge to the healthcare system because of its high prevalence, morbidity and mortality. The pending increase in prevalence due to the ageing population and the relatively poor prognosis of heart failure highlight the necessity to further optimise the organisation of heart failure care [1, 2]. Strategies to enhance the coordination between primary, secondary and tertiary healthcare workers may improve clinical outcome and reduce heart failure associated costs.

In the Dutch Alphen-Leiderdorp-Leiden region, collaboration between general practitioners and cardiologists was recently initiated aiming to develop a regional all-phase integrated heart failure care program. A survey among general practitioners in Alphen and heart failure patients of the Alrijne Hospital revealed that patients preferred to receive care from the heart failure clinic, but they wanted their general practitioner to be more involved. General practitioners would appreciate to expand their involvement in heart failure care. Subsequently, a working group was created consisting of cardiologists, heart failure nurses and primary healthcare workers that developed four protocols aiming to streamline patient flow from primary to speciality care and vice-versa. All protocols are in line with the most recent European Society of Cardiology heart failure guideline [3]. The first protocol stipulates the diagnostic measures to be taken by the general practitioner and cardiologist when heart failure is suspected. If the diagnosis is established, the cardiologist identifies the underlying cardiac problem, which is crucial for therapeutic reasons, as the precise pathology determines the specific treatment. The available diagnostic and therapeutic modalities are outlined in the second protocol, which was developed in collaboration with the Leiden University Medical Centre (LUMC). The third protocol describes the seamless transition to long-term management of stable patients. The general practitioner is the main caregiver in stable patients. However, several circumstances necessitate persistent (co)-treatment by a cardiologist in a secondary hospital or in a tertiary referral hospital. The fourth protocol describes the organisation of care for end-stage heart failure patients. The general practitioner mainly delivers palliative care and hospital admissions are prevented. Specific attention is paid to temporary deactivation of implantable cardioverter-defibrillator therapy. The protocols are merged in one flowchart (Fig. 1) to emphasise the all-phase integrated clinical framework for diagnostic and therapeutic decision making in heart failure patients.

**Fig. 1:**
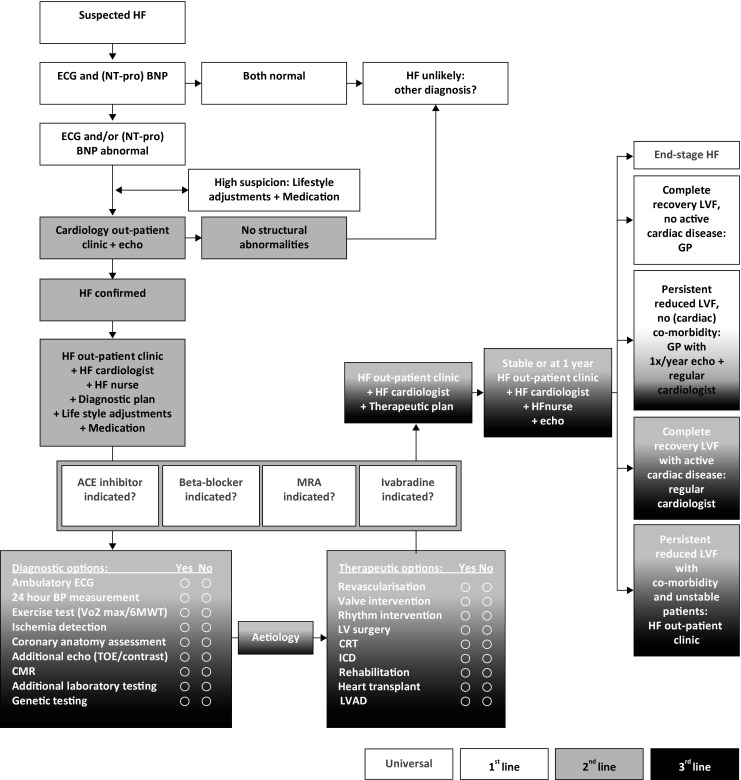
Flowchart for all phase integrated diagnostic and therapeutic decision making in heart failure. *ACE* angiotensin-converting enzyme, *(NT-pro) BNP* (N-terminal pro) brain natriuretic peptide, *BP* blood pressure, *CMR* cardiac magnetic resonance, *CRT* cardiac resynchronisation therapy, *ECG* electrocardiography, *HF* heart failure, *GP* general practitioner, *ICD* implantable cardioverter-defibrillator, *LV* left ventricular, *LVAD* left ventricular assist device, *LVF* left ventricular function, *MRA* mineral receptor antagonist, *TOE* transoesophageal echocardiogram, *6MWT* 6-min walk test

Co-operation between primary, secondary and tertiary healthcare providers may optimise heart failure care. By working together, tailored care in adherence with the guidelines can be provided as close to home as possible. The current framework complies with the recent recommendations of the World Heart Failure Alliance since it provides a system that delivers timely access to diagnostic services and treatment of heart failure, as well as a seamless transition to long-term care [2]. The concept that a standardised regional program can improve care has already been established in acute infarction patients [4] and resulted in the Dutch national project ‘Connect acute infarction’. Potentially, the presented framework may be used as a start-up for a new national project ‘Connect heart failure’, which may offer a prospective solution to the increasing demand for heart failure care.

## Sources of funding

This research received no funding.

## Conflict of interest

The Department of Cardiology of the LUMC receives research grants from Biotronik, Boston Scientific and Medtronic.
